# Redox-active compounds with a history of human use: antistaphylococcal action and potential for repurposing as topical antibiofilm agents

**DOI:** 10.1093/jac/dku409

**Published:** 2014-11-03

**Authors:** N. Ooi, E. A. Eady, J. H. Cove, A. J. O'Neill

**Affiliations:** 1 Antimicrobial Research Centre and School of Molecular and Cellular Biology, University of Leeds, Leeds, UK; 2 Harrogate and District NHS Foundation Trust, Harrogate, UK

**Keywords:** *Staphylococcus aureus*, *Staphylococcus epidermidis*, biofilm, antibacterial, mode of action

## Abstract

**Objectives:**

To investigate the antistaphylococcal/antibiofilm activity and mode of action (MOA) of a panel of redox-active (RA) compounds with a history of human use and to provide a preliminary preclinical assessment of their potential for topical treatment of staphylococcal infections, including those involving a biofilm component.

**Methods:**

Antistaphylococcal activity was evaluated by broth microdilution and by time–kill studies with growing and slow- or non-growing cells. The antibiofilm activity of RA compounds, alone and in combination with established antibacterial agents, was assessed using the Calgary Biofilm Device. Established assays were used to examine the membrane-perturbing effects of RA compounds, to measure penetration into biofilms and physical disruption of biofilms and to assess resistance potential. A living skin equivalent model was used to assess the effects of RA compounds on human skin.

**Results:**

All 15 RA compounds tested displayed antistaphylococcal activity against planktonic cultures (MIC 0.25–128 mg/L) and 7 eradicated staphylococcal biofilms (minimum biofilm eradication concentration 4–256 mg/L). The MOA of all compounds involved perturbation of the bacterial membrane, whilst selected compounds with antibiofilm activity caused destructuring of the biofilm matrix. The two most promising agents [celastrol and nordihydroguaiaretic acid (NDGA)] in respect of antibacterial potency and selective toxicity against bacterial membranes acted synergistically with gentamicin against biofilms, did not damage artificial skin following topical application and exhibited low resistance potential.

**Conclusions:**

In contrast to established antibacterial drugs, some RA compounds are capable of eradicating staphylococcal biofilms. Of these, celastrol and NDGA represent particularly attractive candidates for development as topical antistaphylococcal biofilm treatments.

## Introduction

Biofilms comprise surface-attached microbial communities encased within a self-produced extracellular matrix and are found in ∼80% of bacterial infections in humans.^1^ Infections including a biofilm component can be extremely challenging to treat: not only are biofilms refractory to killing by the majority of antibacterial drug classes in clinical use, but they also represent a sanctuary site in which bacteria are physically shielded from attack by the host immune system.^1,2^ To address the current difficulties in treating biofilm infections, it will be important to discover antibacterial agents capable of demonstrating effective killing and/or eradication of bacterial biofilms.

In ongoing studies, we have been seeking to identify compounds with the potential for topical treatment of human skin infections including a biofilm component, such as infected wounds. Our approach has focused on evaluating the antibiofilm activity of compounds already in human use for cosmetic or healthcare applications, or that have been approved for human consumption, since the repurposing of chemicals with established safety profiles potentially offers an accelerated route by which antibiofilm agents can enter into clinical use. We recently reported that *tert*-butylhydroquinone (TBHQ), an antioxidant with a history of safe use as a food preservative, undergoes spontaneous conversion to *tert*-butylbenzoquinone (TBBQ), which exhibits potent activity against staphylococcal biofilms.^3^ In the present study, we examined the antistaphylococcal activity of a range of other redox-active compounds with a history of safe or traditional use in humans, with an emphasis on assessing their activity against biofilms and elucidating their antibacterial and antibiofilm mode(s) of action. Several of the compounds investigated displayed potent activity against staphylococcal biofilms and a subset of these was found to exhibit properties that make them suitable to be considered for application as topical biofilm treatments. These compounds, or derivatives thereof, may therefore represent candidates for development as novel therapies for superficial skin infections.

## Materials and methods

### Bacterial strains and routine culture

The laboratory strain *Staphylococcus aureus* SH1000^4,5^ and the prolific biofilm-forming strains *S. aureus* UAMS-1^6^ and *Staphylococcus epidermidis* RP62A (ATCC 35984)^7^ were used throughout this study. Bacteria were cultured using Mueller–Hinton broth (MHB) and agar (MHA) (Oxoid, Cambridge, UK), supplemented with calcium (50 mg/L, in the form of CaCl_2_) for studies with daptomycin.

### Chemicals and reagents

The compounds used in this study, 2,2′-methylenebis[6-*tert*-butyl-4-methylphenol] (AO 2246), bakuchiol, benzoyl peroxide, carnosic acid, celastrol, dihydroxychalcone, 8-hydroxyquinoline, idebenone, dodecyl gallate (lauryl gallate), menadione, nordihydroguaiaretic acid (NDGA), thymohydroquinone, thymoquinone, totarol and vitamin K5 hydrochloride, were gifts from Syntopix Group plc (Bradford, UK) [known latterly as Evocutis plc (Wetherby, UK)]. Other chemicals and antibiotics were from Sigma-Aldrich (Poole, UK), with the following exceptions: ampicillin (Fisher Scientific, Loughborough, UK), cefotaxime (MP Biomedicals, Illkirch Cedex, France), ciprofloxacin (Bayer, Leverkusen, Germany), daptomycin (Cubist Pharmaceuticals, Lexington, MA, USA), flucloxacillin (CP Pharmaceuticals, Wrexham, UK), meropenem (AstraZeneca, Wilmington, DE, USA), vancomycin (Duchefa Biochemie, Haarlem, the Netherlands) and ethanol (Fisher Scientific). SYPRO^®^ Ruby, DiSC_3_(5) and the Live/Dead *Bac*Light™ kit were from Invitrogen (Paisley, UK).

### Evaluation of antibacterial activity

Antibacterial susceptibility testing with planktonic cultures was performed by exposing bacteria to serial dilutions of compounds in MHB according to the broth microdilution guidelines provided by the CLSI.^8^ Minimum biofilm eradication concentrations (MBECs) were determined for biofilm cultures grown using the Calgary Biofilm Device (CBD) (Nunc A/S, Roskilde, Denmark) and were defined as the lowest concentration of compound capable of sterilizing the biofilm.^9^

Synergistic interactions between redox-active compounds and established antibacterial drugs were examined against biofilms grown on the CBD using the chequerboard method, as described previously.^10^ A fractional inhibitory concentration (FIC) index of ≤0.5 was taken to indicate a synergistic interaction, whilst values of 1 and ≥2 were taken to indicate additivity and antagonism, respectively.^10^

Time–kill experiments with exponential-phase cultures were performed as described^3^ using cultures grown to an OD_600_ of 0.2 (∼10^8^ cfu/mL). For time–kill studies with non-growing (stationary phase) bacteria, overnight (∼16 h) cultures of *S. aureus* SH1000 were centrifuged and cells resuspended in the spent medium to an OD_600_ of 0.2 prior to exposure to antibacterial agents. Persister cells were generated by growing *S. aureus* SH1000 to an OD_600_ of 0.2 and exposing the cells to ampicillin or ciprofloxacin at 10× MIC for 24 h at 37°C. Persisters were washed, resuspended in the same volume of fresh MHB and challenged with antibacterial compounds at 10× MIC.^11,12^ Bacterial viability was monitored post-challenge by plating cultures onto MHA and enumerating colonies after incubation for 18–24 h at 37°C. To detect bacterial lysis following challenge with redox-active compounds at 4× MIC, the culture turbidity of early exponential-phase cultures (OD_600_ of 0.2) at 37°C was monitored by absorbance measurements at 600 nm.^13^

### Antibacterial mode of action (MOA) studies

The effect of 10 min of exposure to antibacterial compounds on the integrity of the staphylococcal membrane was assessed at 4× MIC using the *Bac*Light™ assay^14^ and by monitoring leakage of potassium ions from cells resuspended in 5 mM HEPES-glucose buffer (pH 7.2) after 3 h.^15,16^ Membrane potential was determined by measuring intracellular and extracellular levels of the fluorescent dye DiSC_3_(5) following exposure to antibacterial agents in HEPES-glucose buffer for 3 h at 4× MIC.^15,17^ The effect of compounds on mammalian membranes was examined by monitoring haemolysis of erythrocytes isolated from lithium heparin-treated whole equine blood (Matrix Biologicals Ltd, Hull, UK), as previously described.^3^

### Assessing penetration of redox-active compounds into staphylococcal biofilms

Biofilms were grown on cellulose ester membrane filter discs (Millipore, Billerica, MA, USA) placed on Brain Heart Infusion Agar (BHIA; Oxoid) for 48 h,^7^ and the discs were then transferred to BHIA containing redox-active compounds. A 13 mm cellulose disc (Millipore) was placed on the biofilm, on top of which was placed a 6 mm filter paper disc (Oxoid) moistened with PBS. Following incubation at 37°C for 24 h, the 6 mm disc was transferred to MHA spread with SH1000. After incubation at 37°C for 24 h, the diameter of the zone of inhibition around the disc was measured and compared with a calibration curve of zones of inhibition generated using discs impregnated with known concentrations of the test compound. Percentage penetration of compound into the biofilm was calculated with respect to a control assembly containing no biofilm.^18^

### Determining the effect of redox-active agents on biofilm structure and viability

Alterations in biofilm structure following challenge with antibacterial agents were assessed by quantifying matrix material and adhered cells by staining with SYPRO^®^ Ruby and SYTO^®^ 9, respectively.^19^ Microtitre plates were pre-conditioned with 20% normal pooled human plasma (Sera Laboratories International, Haywards Heath, UK) in 0.05 M carbonate buffer overnight at 4°C. Wells were seeded with *S. aureus* SH1000 in Tryptone Soya Broth (Oxoid) and plates were incubated for 24 h at 37°C with gentle shaking to establish biofilms. Biofilms were then challenged with redox-active compounds at 256 mg/L in MHB or with proteinase K (100 mg/L) in buffer (20 mM Tris, pH 7.5, and 100 mM NaCl) for 60 min or 24 h.^19^ Biofilms were washed in water before being stained with undiluted FilmTracer™ SYPRO^®^ Ruby containing 0.17 μM SYTO^®^ 9 for 30 min. Following a further wash in water, fluorescence was measured at an excitation wavelength of 480 nm and an emission wavelength of 620 nm (matrix) or 520 nm (cells).^19^ In parallel experiments, total biofilm viability was measured following exposure of established biofilms to compounds for 1 h. Detached cells were collected and adherent cells were dispersed by incubation with proteinase K (100 mg/L) in buffer for 1 h. All cells were washed in PBS before being plated onto MHA and enumeration of colonies was carried out after incubation for 18–24 h at 37°C.

### Preliminary evaluation of the potential for use as topical antistaphylococcal agents

To examine whether redox-active compounds are toxic to human skin, the effect of compounds on a human living skin equivalent was assessed. Fully differentiated, 28 day old LabSkin™ (Innovenn, Dublin, Ireland)^20^ and maintenance medium were produced and donated by Evocutis plc. Skin was exposed to 100 μL of test compound at 10× or 4× MIC in sterile deionized water (solvent load: 0.2% ethanol, v/v) for 24 h at 37°C, 5% CO_2_, >95% relative humidity.^20^ Drug-free controls were exposed to deionized water or solvent alone and the positive control was incubated with 5% SDS. Following incubation, LabSkin™ medium was sampled and tissue was fixed in 10% neutral buffered formalin for 24 h. Fixed tissues were embedded in wax and subjected to haematoxylin/eosin staining and visual inspection of tissue sections.^20^ To detect potential skin irritation induced by application of compounds, IL-1α was measured in medium sampled following 24 h of incubation with compounds.^21,22^ IL-1α quantification was performed using a human IL-1α/IL-1F1 Quantikine ELISA Kit (R&D Systems, Abingdon, UK) according to the manufacturer's instructions and compared with the positive control.

The potential for development of resistance to redox-active compounds was assessed by determining mutation frequencies at 4× MIC,^7^ and selection using the extended gradient MIC method as previously described.^23^

## Results and discussion

### Antistaphylococcal properties of redox-active compounds

Although it has previously been reported that the redox-active compounds examined in this study possess antibacterial activity,^24–38^ most have not been evaluated for their antistaphylococcal activity using standardized procedures for susceptibility testing. Furthermore, these compounds have not been evaluated for their ability to eradicate staphylococcal biofilms. Since staphylococci form biofilms that vary in their matrix composition,^7,39,40^ susceptibility testing was carried out with three staphylococcal strains (*S. aureus* SH1000 and UAMS-1, *S. epidermidis* RP62A) that form distinct types of biofilm. Using CLSI methodology, the 15 redox-active compounds tested exhibited MICs ranging from 0.25 to 128 mg/L for planktonic cultures of *S. aureus* and *S. epidermidis* (Table 1). In subsequent experiments to assess their antibiofilm activity, approximately half (7/15) of these compounds demonstrated complete eradication of preformed staphylococcal biofilms at concentrations ≤256 mg/L, in some cases exhibiting MBEC values as low as 4 mg/L (Table 1). By contrast, none of the established antibacterial agents tested (cefotaxime, chlorhexidine, ciprofloxacin, cetyltrimethylammonium bromide, daptomycin, erythromycin, fosfomycin, flucloxacillin, fusidic acid, gentamicin, meropenem, mupirocin, oxacillin, rifampicin, SDS, tetracycline and vancomycin) was able to eradicate SH1000 biofilms at ≤256 mg/L (data not shown).

**Table 1.** MICs and MBECs of redox-active compounds for staphylococci

Compound	Strain					
	SH1000	UAMS-1	RP62A	SH1000	UAMS-1	RP62A
	MIC (mg/L)			MBEC (mg/L)		
AO 2246	4	4	4	16	16	128
Bakuchiol	4	4	4	16	8	8
Benzoyl peroxide	64	32	32	256	32	64
Carnosic acid	32	32	8	128	64	64
Celastrol	1	0.5	0.25	32	4	4
Dihydroxychalcone	128	128	128	>256	>256	>256
8-Hydroxyquinoline	4	2	1	>256	32	>256
Idebenone	64	64	32	>256	256	>256
Lauryl gallate	64	64	16	>256	256	>256
Menadione	16	8	8	>256	128	256
NDGA	64	64	64	128	16	256
Thymohydroquinone	16	8	16	>256	16	64
Thymoquinone	16	8	8	>256	4	32
Totarol	4	2	2	16	8	16
Vitamin K5 hydrochloride	32	16	16	>256	64	128

**Figure 1.**
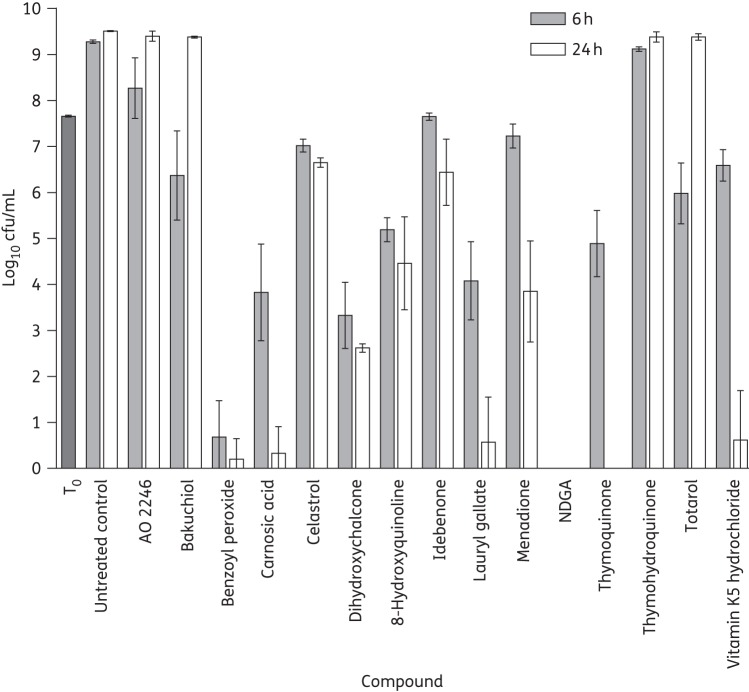
Evaluation of the bactericidal activity of redox-active compounds at 4× MIC for *S. aureus* SH1000. T_0_ represents the number of cells present in cultures prior to addition of compounds. Results are means of at least three independent replicates and error bars show standard deviations.

The ability of several of the redox-active compounds to completely eradicate biofilms suggested that they exert a bactericidal action. To assess this, we examined compound-mediated killing of SH1000 in planktonic culture by viable counting. At 4× MIC, the majority of the compounds (benzoyl peroxide, carnosic acid, dihydroxychalcone, 8-hydroxyquinoline, lauryl gallate, menadione, NDGA, thymoquinone and vitamin K5 hydrochloride) were bactericidal (Figure 1). At concentrations corresponding to the MBEC, benzoyl peroxide, carnosic acid, celastrol and NDGA sterilized planktonic cultures at 24 h (limit of detection of 1 log_10_ cfu/mL). AO 2246, bakuchiol and totarol killed bacteria initially, causing ≥2.8 log_10_ reduction in cfu/mL after 1 h, but allowed grow-back over a 24 h period (data not shown). Therefore, none of the agents with antibiofilm activity displayed exclusively bacteriostatic properties. Bacterial killing was not associated with reductions in culture turbidity over 24 h, indicating that killing did not result from, or trigger, cell lysis (data not shown); however, benzoyl peroxide interfered with absorbance readings at OD_600_ and was not therefore evaluated for its ability to cause bacterial lysis.

### MOA of redox-active compounds

**Figure 2.**
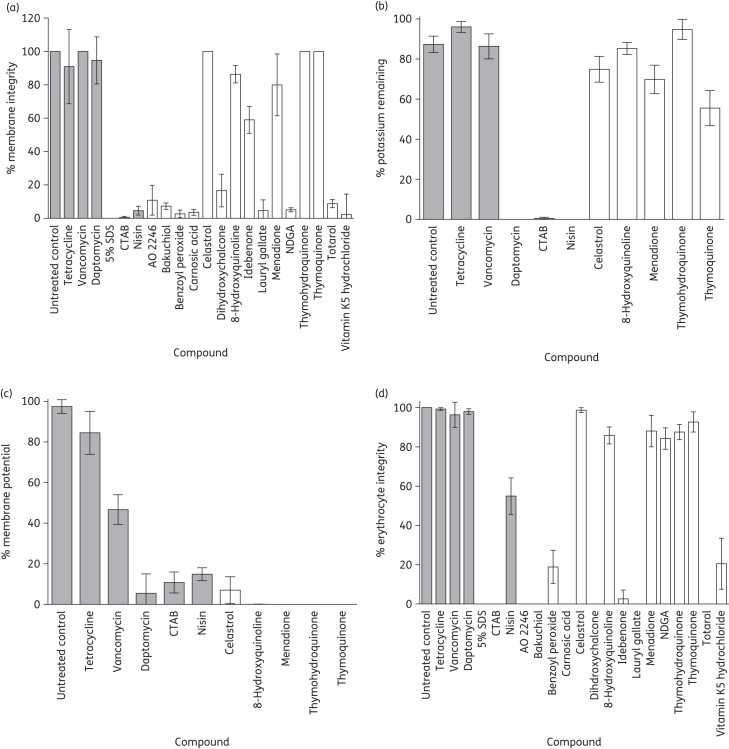
Effect of redox-active compounds and comparator agents at 4× MIC on bacterial and mammalian membranes. Bacterial membrane integrity was measured using the *Bac*Light™ assay (a) and by monitoring leakage of intracellular potassium (b). Bacterial membrane potential was measured by leakage of DiSC_3_(5) (c). Mammalian membrane integrity was measured by leakage of haemoglobin from erythrocytes (d). Filled bars show comparator agents. Results are means of at least three independent determinations and error bars show standard deviations.

The results of previous studies by us and others suggest that the redox-active compounds TBBQ, bakuchiol and totarol exert their antibacterial effects through perturbation of the bacterial membrane.^3,35,41^ We therefore examined whether other redox-active compounds act in the same manner. At 4× MIC, 10 of the 15 compounds compromised the integrity of the membrane of *S. aureus* SH1000 within 10 min, as measured by the *Bac*Light™ assay (Figure 2a). Since this assay may not reliably detect subtle membrane perturbation (e.g. loss of membrane potential) or perturbation occurring over longer time periods, we employed additional assays to assess whether the other five compounds also mediate membrane perturbation. All five caused complete or near-complete loss of membrane potential within 3 h at 4× MIC, as measured by reduction in intracellular and increase in extracellular levels of the membrane-potential sensitive dye DiSC_3_(5); furthermore, some of these agents (e.g. thymoquinone) prompted leakage of intracellular potassium over this duration and at this concentration (Figure 2b and c). Thus, all of the redox-active compounds tested caused membrane perturbation, albeit at different rates and to different extents.

To establish whether the redox-active compounds display specificity for the bacterial membrane, we challenged mammalian erythrocytes with compounds at a concentration equivalent to 4× MIC for *S. aureus* SH1000 and monitored haemoglobin leakage over 1 h. Of the 15 agents tested, 6 (celastrol, 8-hydroxyquinoline, menadione, NDGA, thymohydroquinone and thymoquinone) did not induce substantial haemolysis (Figure 2d), suggesting that they exert selectively toxic effects on bacterial membranes. We focused our subsequent studies on celastrol and NDGA, since the selectivity of these redox-active compounds for bacterial membranes and their ability to eradicate staphylococcal biofilms suggested the greatest potential for therapeutic utility.

### Mechanistic basis for antibiofilm activity of redox-active compounds

Since all of the redox-active compounds displayed antibacterial activity against planktonic cells, we sought to examine why some of these agents were able to eradicate staphylococcal biofilms (‘eradicators’), whilst others were not (‘non-eradicators’). We studied a representative compound of each class (the eradicator celastrol and the non-eradicator 8-hydroxyquinoline) and examined whether lack of antibiofilm activity was due to a failure of compounds to penetrate the biofilm.^42^ The non-eradicator 8-hydroxyquinoline showed more extensive biofilm penetration (105 ± 2%) than the eradicator celastrol (44 ± 4%). Therefore, failure of compounds to penetrate the biofilm does not appear to underlie the lack of activity of the non-eradicators against biofilms.

**Figure 3.**
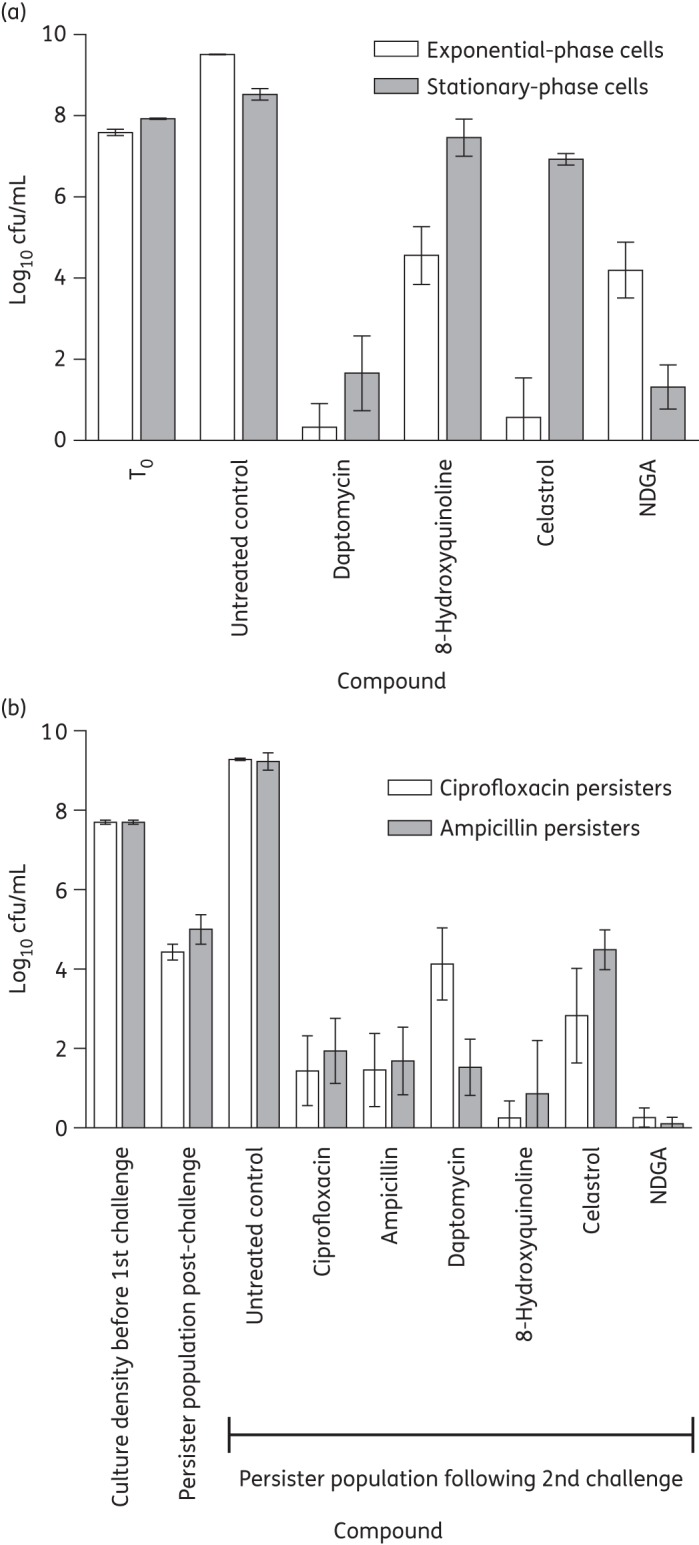
Viability of *S. aureus* SH1000 slow-growing and persister cells following exposure to compounds for 24 h. (a) Exponential- and stationary-phase cell viability following exposure to biofilm eradicators at the MBEC or biofilm non-eradicators at 256 mg/L. T_0_ is the culture density before the addition of compounds. (b) Cell viability of ciprofloxacin and ampicillin persisters following exposure to compounds at 10× MIC. Results are means of at least three independent determinations and error bars show standard deviations.

The failure of most established antibacterial drugs to eradicate bacterial biofilms has been attributed to the inability of these compounds to effectively kill the large population of slow- or non-growing (SONG) bacteria, including persister cells, present in biofilms.^43^ To assess whether redox-active compounds capable of biofilm eradication do so because they retain bactericidal activity against SONG cells, we examined their ability to kill stationary-phase and persister cells in planktonic culture. Neither celastrol nor NDGA showed significantly enhanced killing of SONG cells compared with compounds lacking antibiofilm activity (daptomycin and 8-hydroxyquinoline) (Figure 3).

**Figure 4.**
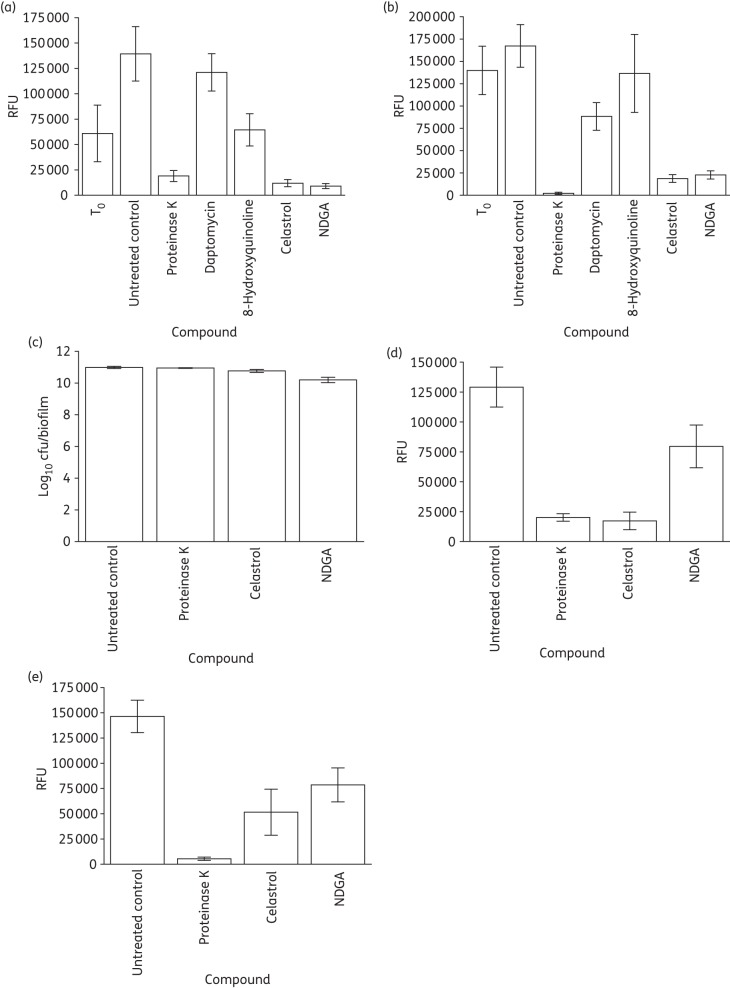
Cell viability and quantification of adherent material by fluorescent staining following exposure of biofilms to compounds at 256 mg/L for 24 h or 60 min. (a) Attached matrix (24 h). (b) Attached cells (24 h). (c) Total biofilm cell viability (60 min). (d) Attached matrix (60 min). (e) Attached cells (60 min). The data labelled T_0_ show the quantity of matrix and cells before the addition of compounds and RFU denotes relative fluorescence units. Results are means of at least three independent determinations, with error bars showing standard deviations.

We subsequently examined whether biofilm-eradicating redox-active compounds might act by causing physical perturbation of the biofilm. Biofilm matrix material and cells were quantified following exposure to compounds for 24 h to identify whether compound-induced alterations in biofilm structure distinguished biofilm eradicators from non-eradicators. At 256 mg/L, eradicators significantly reduced the amount of adherent matrix and cells in comparison with non-eradicators (Figure 4a and b). To investigate whether this was a cause or a result of eradicator-mediated perturbation of the biofilm, viable cells and adherent matrix material were quantified following exposure to NDGA and celastrol for a shorter time period (1 h). Over this time period, the compounds had little or no effect on total cell viability (both released and attached cells) (Figure 4c), while adherent material was significantly reduced (Figure 4d and e), implying that biofilm detachment is not simply a consequence of cell death. Therefore, celastrol and NDGA appear to exert their antibiofilm effects at least in part by inducing destructuring of the biofilm.

### Preliminary evaluation of the potential for use of celastrol and NDGA as topical antistaphylococcal agents

For a compound to be developed as a topical antibiofilm agent, it should not cause damage or irritation to the skin upon application. NDGA and celastrol have previously shown no notable adverse effects on human or mouse skin by visual inspection when investigated as topical preparations at 300 mg/L for the treatment of psoriasis and 500 mg/L for reducing inflammation, respectively.^44,45^ Therefore, whilst some evaluation of toxicity has been conducted with both celastrol and NDGA, the possibility that these compounds could trigger irritation and/or physical damage to human skin has not been examined in detail.

**Figure 5.**
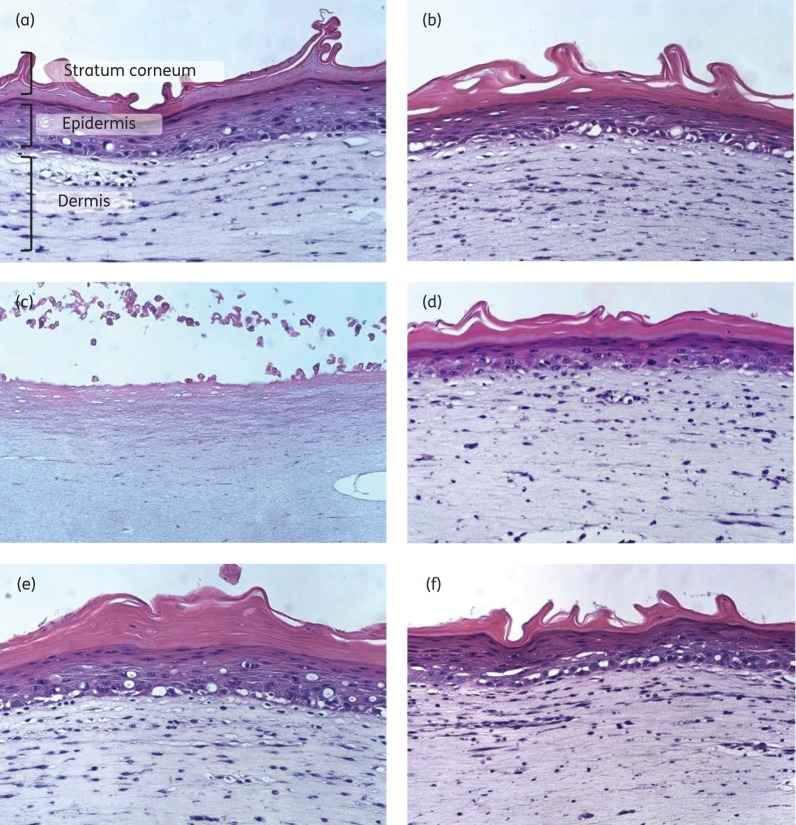
Haematoxylin/eosin-stained sections of LabSkin™ exposed to compounds for 24 h. (a) Untreated control. (b) Solvent (0.2% ethanol, v/v). (c) SDS (5%, w/v). (d) Mupirocin at 10× MIC (0.000125%, w/v). (e) Celastrol at 10× MIC (0.001%, w/v). (f) NDGA at 4× MIC (0.0256%, w/v).

To determine whether celastrol and NDGA exhibit sufficient selective toxicity to allow their topical use on human skin, we examined their effects upon a human living skin equivalent model (LabSkin™). *In vitro*, three-dimensional skin models such as this represent an established means of evaluating the acute dermal toxicity of test compounds.^22,46^ Following 24 h of exposure to either celastrol or mupirocin (control) at 10× *S. aureus* SH1000 MIC (10 and 1.25 mg/L, respectively) we detected no increase in release of the inflammatory marker IL-1α, an indicator of irritation (data not shown).^47^ The limited solubility of NDGA meant that, in order to keep the solvent load below the level (0.5%) causing damage to LabSkin™, the compound could only be tested at a concentration equivalent to 4× *S. aureus* SH1000 MIC (256 mg/L). However, no increase in IL-1α release was detected at this concentration. Haematoxylin/eosin staining of tissue sections following 24 h of exposure to mupirocin, celastrol and NDGA showed no visible detrimental effects on skin (Figure 5d–f). By contrast, exposure of LabSkin™ to the known irritant SDS induced a 30-fold increase in release of IL-1α (data not shown) and was severely damaging to the skin structure, causing shedding of the top layers of skin (stratum corneum and epidermis) and injury to the dermis (Figure 5c). Thus, celastrol and NDGA did not cause irritation or damage to fully differentiated skin at concentrations above those required to inhibit growth of bacterial cultures.

**Figure 6.**
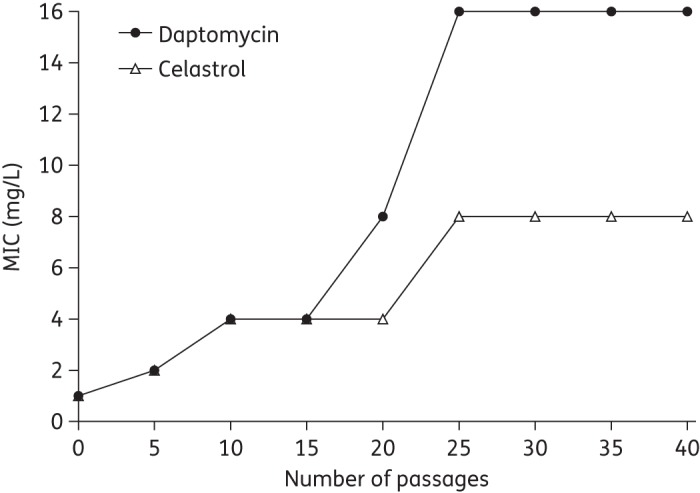
Selection of daptomycin- and celastrol-resistant mutants of *S. aureus* SH1000. Results are representative of experiments carried out on eight independent occasions using the extended gradient MIC method.^23^

A desirable feature of any potential antibacterial agent for clinical use is a low propensity to select resistance. The resistance potential for NDGA and celastrol was initially evaluated by plating saturated cultures of *S. aureus* SH1000 onto agar containing these compounds at 4× MIC. In neither case were resistant mutants isolated (mutation frequency <5.0 × 10^−9^). This suggested that the redox-active compounds have low resistance potentials and that multiple mutations may be required to develop resistance to the agents.^48^ To examine whether resistance might arise following prolonged selection, we employed the extended gradient MIC method,^23^ using an established antibacterial agent with a low resistance potential (daptomycin) as a comparator.^24^ Resistance (defined for the purpose of this experiment as a ≥4-fold increase in MIC) to daptomycin arose in eight independent selection cultures of *S. aureus* SH1000 after 10 passages and the most resistant strain recovered displayed a 16-fold reduction in daptomycin susceptibility following 40 passages (Figure 6). Celastrol resistance arose over the same time-scale as daptomycin resistance (10 passages) in all selection cultures and the most resistant strain exhibited an 8-fold reduction in celastrol susceptibility after 40 passages (Figure 6). No reduction in NDGA susceptibility was observed in *S. aureus* SH1000 after 40 passages in the presence of the compound (data not shown). Thus, both celastrol and NDGA have low resistance potential, implying that resistance would be unlikely to arise rapidly should these compounds be used in the clinical setting for topical treatment of staphylococcal infection.

Previous studies with a subset of the agents under investigation here have reported compound-mediated potentiation of the antistaphylococcal activity of established antibacterial drugs. Thus, dihydroxychalcone showed synergism with doxycycline, gentamicin and ciprofloxacin,^49^ and totarol increased the activity of β-lactams against *S. aureus*.^34^ Therefore, we investigated the possibility that celastrol and NDGA might exhibit synergistic effects in biofilm eradication assays when combined with licensed antibacterial drugs (tetracycline, ciprofloxacin, erythromycin, gentamicin and oxacillin). Celastrol and NDGA both exhibited synergy with gentamicin against *S. aureus* SH1000 biofilms (FIC index ≤0.25 and ≤0.15, respectively), an antibiotic that was alone unable to eradicate biofilms at the concentrations tested (≤256 mg/L), whilst all the other compound–antibacterial drug combinations showed additive effects (FIC index >0.5). Aminoglycosides are often applied as a topical cream at 0.1% (1 g/L) for the sterilization of infected wounds,^50^ but this concentration of gentamicin is insufficient to eradicate established staphylococcal biofilms, at least *in vitro* (data not shown). We therefore investigated the concentrations of celastrol or NDGA that would need to be administered alongside 0.1% gentamicin to eradicate preformed staphylococcal biofilms *in vitro*. Eradication of *S. aureus* SH1000 biofilms by 0.1% gentamicin was achieved in conjunction with 0.004 mg/L celastrol or 0.25 mg/L NDGA. Our results suggest that the combination of gentamicin with low concentrations of these redox-active compounds could potentially be employed topically to more effectively treat superficial staphylococcal infections that involve a biofilm component.

### Conclusions

We have characterized a number of redox-active compounds that, in addition to demonstrating antistaphylococcal activity, are capable of eradicating established staphylococcal biofilms. A subset of these compounds shows a degree of prokaryotic selectivity. The antibacterial action of these compounds is mediated by membrane perturbation, whilst the antibiofilm MOA appears to involve disruption of the biofilm matrix. Neither celastrol nor NDGA caused irritation or damage to the skin surface in a living skin equivalent model. Therefore, these compounds deserve further consideration as potential antibiofilm agents for topical application, either alone or in a synergistic combination with aminoglycosides. The low resistance potential of both celastrol and NDGA suggests that their use as topical antibiofilm treatments would not rapidly become compromised by the development of resistance.

## Funding

This work was supported by BBSRC Industrial CASE studentship BB/G017158/1 in conjunction with Syntopix Group plc (known latterly as Evocutis plc).

## Transparency declarations

E. A. E. and J. H. C. are former employees of Syntopix Group plc (known latterly as Evocutis plc). N. O. and A. J. O.: none to declare.
